# Identifying the regulatory cascade governing amyloid precursor protein macropinocytosis and subsequent production of amyloid‐beta

**DOI:** 10.1002/alz.092015

**Published:** 2025-01-03

**Authors:** Jordan M Krupa, Abdul Moiz Naqvi, Adrianna R Tsang, Claudia Seah, Stephen H Pasternak

**Affiliations:** ^1^ University of Western Ontario, London, ON Canada; ^2^ Robarts Research Institute, London, ON Canada; ^3^ Parkwood Institute/St Joseph’s Healthcare Center, London, ON Canada

## Abstract

**Background:**

Previously, we identified macropinocytosis as a novel mechanism for direct and rapid trafficking of cell surface APP to lysosomes, bypassing early and late endosomes. This process depends on the activity of Arf6 and several Rho‐GTPases, and inhibition of macropinocytosis reduces amyloid‐beta (Aβ) production. Macropinocytosis is relatively unstudied in neurons and neuronal cells. Based on data from neuronal and non‐neuronal cell types, we hypothesized that the crosslinking of APP at the cell surface recruits Fe65, which acts as a scaffold for the recruitment and activation of Arf6. Arf6 then recruits the Rho‐GTPases Rac1, Cdc42 and RhoA, resulting in APP internalization by macropinocytosis and its cleavage to Aβ within the lysosome.

**Methods:**

Neuro2a (N2a) neuronal cells or human IPSC‐derived primary cortical neurons were transduced with fluorescent‐tagged Fe65, Arf6, Rac1, Cdc42 or RhoA. Membrane PI(4,5)P2 was used to mark membrane ruffling using fluorescent‐tagged PLCδ‐PH. Live neuronal cells or neurons were incubated with anti‐APP antibodies on ice to crosslink cell surface APP. Cells were incubated at 37^°^C and imaged at specific time points using confocal microscopy and live cell imaging.

**Result:**

Crosslinking APP resulted in rapid recruitment of Fe65 and Arf6 to APP and membrane ruffles within 30 sec, which rapidly decreased by 2 min. The Rho‐GTPases Rac1, Cdc42 and RhoA demonstrated sustained colocalization with APP and ruffles from 30 sec through to 2 min. This decreased at 5 and 10 min as APP was internalized. The inhibition of Arf6 using NAV2729 prevented the recruitment of itself, Rac1, Cdc42, and RhoA at 30 sec. However, inhibition of Rac1 by EHT 1864 had no significant effect on the recruitment of Rac1 to crosslinked APP.

**Conclusion:**

These results demonstrate the sequential recruitment of regulatory proteins which regulate macropinocytosis of APP in response to its crosslinking. Fe65 and Arf6 are rapidly recruited to APP, followed by sustained recruitment of Rac1, RhoA and Cdc42. These regulatory proteins could be targeted to modulate APP‐lysosomal trafficking and reduce Aβ production.

